# Agroforestry Management Systems Drive the Composition, Diversity, and Function of Fungal and Bacterial Endophyte Communities in Theobroma Cacao Leaves

**DOI:** 10.3390/microorganisms8030405

**Published:** 2020-03-13

**Authors:** Franziska Wemheuer, Dirk Berkelmann, Bernd Wemheuer, Rolf Daniel, Stefan Vidal, Hervé Bertin Bisseleua Daghela

**Affiliations:** 1 Section of Agricultural Entomology, Department of Crop Sciences, University of Göttingen, Grisebachstr. 6, D-37077 Göttingen, Germany; fwemheu@gwdg.de (F.W.); hbissel@gmail.com (H.B.B.D.); 2 Genomic and Applied Microbiology & Göttingen Genomics Laboratory, Institute of Microbiology and Genetics, University of Göttingen, Grisebachstr. 8, D-37077 Göttingen, Germany; dberkel@gwdg.de (D.B.); bwemheu@gwdg.de (B.W.); rdaniel@gwdg.de (R.D.); 3 Laboratory of Entomology, Institute of Agricultural Research for Development (IRAD), BP 2067, Yaoundé, Cameroon

**Keywords:** microbial diversity, endophytes, core microbiome, metabarcoding, agroforestry management systems, functional predictions, *Theobroma cacao*

## Abstract

Cacao (*Theobroma cacao* L.) is one of the most economically important crops worldwide. Despite the important role of endophytes for plant growth and health, very little is known about the effect of agroforestry management systems on the endophyte communities of *T.* *cacao*. To close this knowledge gap, we investigated the diversity, community composition, and function of bacterial and fungal endophytes in the leaves of *T.* *cacao* trees growing in five major cacao-growing regions in the central region of Cameroon using DNA metabarcoding. Fungal but not bacterial alpha diversity measures differed significantly between the agroforestry management systems. Interestingly, less managed home-garden cacao forests harbored the lowest fungal richness and diversity. Our results suggest that the composition of bacterial and fungal endophyte communities is predominantly affected by agroforestry management systems and, to a lesser extent, by environmental properties. The core microbiome detected comprised important fungal phytopathogens, such as *Lasiodiplodia* species. Several predicted pathways of bacterial endophytes and functional guilds of fungal endophytes differed between the agroforest systems which might be attributed to bacteria and fungi specifically associated with a single agroforest. Our results provide the basis for future studies on foliar fungal and bacterial endophytes of *T.* *cacao* and their responsiveness towards agroforestry management systems.

## 1. Introduction

Cacao (*Theobroma cacao* L.) is one of the most economically important crops worldwide. To accommodate the increasing global demand, cocoa production has increased to approximately 5.2 million tons in 2017 (FAO Statistical Database; http://faostat.fao.org). Cacao trees have been traditionally established under thinned canopies of primary or old secondary forests [1]. To enhance their short-term income, farmers in many parts of the world have converted these shaded cacao systems into non-shaded, high intensive monocultures [1,2]. The conversion of tropical rainforests and agricultural homogenization, however, causes severe problems such as biodiversity loss as well as an increased risk of pest outbreaks [3,4,5]. Cacao agroforestry management systems (AMSs), such as traditional forest gardens, may help to alleviate disease and pest problems [6,7]. Cacao AMSs include numerous cultivated plants (e.g., cocoa and bananas) and natural forest tree species. Additionally, they provide a wide range of benefits, including livelihoods for farmers, as well as the conservation of natural resources [1,8]. Consequently, the relationship among different cacao AMSs and their role in maintaining biodiversity has received more attention during the last years [1,4,9].

Endophytic fungi and bacteria have been found in all plant species investigated to date [10]. An increasing number of studies has assessed the community composition [11,12,13,14] and the diverse functional effects of endophytes on their host plants [15,16]. Beneficial endophytes have been reported to promote plant nutrition acquisition and growth [16,17]. Moreover, endophytes may enhance the resistance of their host plants to plant pathogens [18,19,20]. This is especially important due to the wide range of fungal pathogens attacking cacao trees [21,22]. Several of these pathogens are responsible for severe yield losses [22,23]. As a consequence, the potential of endophytic fungi and bacteria as biocontrol agents of important cacao pathogens such as *Moniliophthora roreri* or *Phytophthora capsici* has been evaluated [12,15,20]. For instance, several endospore-forming bacterial endophytes isolated from *T. cacao* inhibited the cacao pathogens *M. roreri*, *M. perniciosa*, and *P. capsica* in antagonism studies [20]. In addition, eight isolates significant inhibited *P. capsici* lesion formation in detached leaf assays compared to untreated control leaves.

Given the high ecological and economic relevance of bacterial and fungal endophytes, it is crucial to decipher endophyte communities in economically important plant species and their influencing factors. Recent studies have shown that agricultural practices such as cropping system, fertilizer, or fungicide application influenced fungal and bacterial endophyte diversity and/or community structures [24,25,26]. In a previous study on fungal endophyte communities of *Coffea arabica*, region and AMS significantly influenced endophytic communities [13]. To date, it is unclear whether foliar fungal and bacterial endophytes of *T. cacao* trees differ among different AMSs as well.

The goals of the current study were to fill this knowledge gap and to obtain first insights into functional and compositional changes in fungal and bacterial endophytes in leaves of *T. cacao* growing in different AMSs. Furthermore, we aimed to identify fungal and bacterial taxa that were responsive to AMSs. Our study was conducted in five major cacao-growing regions (Obala, Boumnyébel, Bakao, Talba, Kédia) in the Central Region of Cameroon. The regions differed in their AMSs ranging from less extensive to more intensively managed cacao agroforests. Foliar bacterial and fungal endophyte communities were investigated by high-throughput Illumina (MiSeq) sequencing targeting the bacterial 16S rRNA gene and the fungal internal transcribed spacer (ITS), respectively. Additionally, we evaluated the agricultural practices and environmental properties shaping bacterial and fungal communities. To better understand plant–endophyte interactions with respect to AMSs, correlation-based indicator species analyses were performed. Moreover, functional profiles were predicted from obtained 16S rRNA data, and fungal community members were classified with respect to functional guilds. We hypothesized that the AMSs and the prevailing environmental conditions impacted microbial colonization of *T. cacao* leaves and, consequently, microbial community composition.

## 2. Materials and Methods

### 2.1. Study Site and Experimental Design

The study was conducted in five major cacao-growing regions (Obala, Boumnyébel, Bakao, Talba, Kédia) in the humid dense forest region in the Central Region of Cameroon between 4°12′ and 4°30′ N and 10°6′ and 11°15′ E (Table 1, Supplementary Table S1). The altitude varied between 450 and 715 m above sea level. Within the growing regions, 20 cacao plantations in seven sites were selected due to differences in AMSs (Table 1, Supplementary Table S1). These systems were grouped as follows: (1) less managed, home-garden cacao forest (Obala; approximately 30 years old), (2) extensively managed old traditional cacao forest garden (Boumnyébel; approximately 50 years old); (3) extensively managed young traditional cacao forest garden (Bakao; approximately 30 years old); (4) the most intensively managed mature traditional cacao forest garden (Talba; 15–20 years old); and (5) intensively managed young traditional cacao forest garden (Kédia; 8–15 years old). In Obala (sites Nkolobang and Ekabita Essélé), cocoa is grown near houses with a high variety of fruit trees species. In Boumnyébel (sites Pan Makak and Simanya), cocoa is grown under a dense cover of shade tree species. The cacao trees in Talba and Bakao are grown in larger farms and on modified savannah agroecosystems, respectively. In Kédia, cocoa is grown under full sunlight in two villages. For further details on the growing regions, see Bisseleua et al. 2009 [4] and Bisseleua et al. 2008 [7].

### 2.2. Sampling

At least two trees from each cacao plantation were randomly selected for sampling, resulting in eight (Bakao, Kédia) or sixteen trees (Boumnyébel, Obala, Talba) per site (Table 1, Supplementary Table S1). These trees were chosen based on the following criteria: healthy appearance, and overall good physiological and nutritional state. For each tree, three mature leaves with the same age were collected between September and October 2014. The collected leaves showed no obvious disease symptoms, including leaf spots, chlorosis, or other types of pathogen-induced lesions. They were immediately cooled down (below 4 °C) and transported to the laboratory. A total of 25 discs (50 mm^2^) per leaf were stamped out from the apical, middle, and basal region of the leaf. The 75 leaf discs of each tree were pooled prior to DNA extraction. A total of 64 *T. cacao* leaf samples were investigated in this study (Supplementary Material Table S1).

### 2.3. Surface Sterilization and DNA Extraction

Different protocols for surface sterilization of leaf discs were tested using varying incubation times. The protocol giving the best surface sterilization success (no microbial growth for all replicates on the three different media types) was used for further analyses. The final surface sterilization protocol included the following steps: consecutive washing in 70% ethanol for 20 s, 2% sodium hypochlorite for 30 s, 70% ethanol for 20 s, followed by three times immersion in sterilized, distilled diethyl pyrocarbonate (DEPC)-treated water for 10 s. Surface-sterilized leaf discs were subsequently dried on tissue paper. Fresh solutions and separate, sterile collection tubes were used for each sample to avoid cross-contaminations. The surface sterilization of leaf discs was controlled for effectiveness as described previously [27] by placing 5–10 sterile leaf discs per location and 50 μL aliquots of the last washing step onto common laboratory agar plates (malt extract agar (MEA), Luria-Bertani-Agar (LB) and potato dextrose agar (PDA)). The plates were incubated in the dark at approximately 25 °C for at least 3 weeks. No growth of microorganisms was observed. In addition, water from the final washing step was subjected to polymerase chain reaction (PCR) targeting the ITS region and the bacterial 16S rRNA gene. No amplification was detected. These results confirmed that the surface sterilization of all leaf discs was successful in eliminating non-cultivable and cultivable fungal and bacterial epiphytes as well as potential DNA traces from the leaf surfaces.

Total DNA was extracted, employing the peqGOLD Plant DNA Mini kit (Peqlab, Erlangen, Germany; now VWR) according to the manufacturer’s instructions with two modifications described previously [27]. Briefly, all surface-sterilized leaf discs were incubated in lysis buffer at 32 °C for 12 h and subsequently homogenized using ethanol-sterilized pestles. The concentration of DNA extracts was quantified using a NanoDrop ND-1000 spectrophotometer (NanoDrop Technologies, Wilmington, USA). In total, the DNA of 64 *T. cacao* leaf samples was subjected to PCR targeting the bacterial 16S rRNA gene and the fungal ITS region.

### 2.4. Amplification and Sequencing of 16S rRNA Genes

Bacterial 16S rRNA genes were amplified using the forward primer S-D-Bact-0341-b-S-17 (5′-CCT ACG GGN GGC WGC AG-3′; [28]) and the reverse primer S-D-Bact-0785-a-A-21 (5′-GAC TAC HVG GGT ATC TAA TCC-3′; [28]) containing Illumina Nextera adapters for sequencing. The PCR reaction (25 µL) contained 5 µL of five-fold Phusion HF buffer, 200 µM of each of the four deoxynucleoside triphosphates, 4 µM of each primer, 1 U of Phusion high fidelity DNA polymerase (Thermo Scientific, Waltham, MA, USA), and approximately 50 ng of the extracted DNA as a template. The negative controls were performed by using the reaction mixture without a template. The following thermal cycling scheme was used: initial denaturation at 98 °C for 30 s, 30 cycles of denaturation at 98 °C for 15 s, annealing at 53 °C for 30 s, followed by extension at 72 °C for 30 s. The final extension was carried out at 72 °C for 2 min. Each sample was subjected to three independent amplifications. Obtained PCR products per sample were controlled for appropriate size, pooled in equal amounts, and purified using the peqGOLD Gel Extraction kit (Peqlab). The quantification of the PCR products was performed using the Quant-iT dsDNA HS assay kit and a Qubit fluorometer, as recommended by the manufacturer (Thermo Scientific). The DNA samples were barcoded using the Nextera XT-Index kit (Illumina, San Diego, USA) and the Kapa HIFI Hot Start polymerase (Kapa Biosystems, USA). Sequencing was performed at the Göttingen Genomics Laboratory on an Illumina MiSeq Sequencing platform (paired end 2 × 300 bp) using the MiSeq Reagent kit v3, as recommended by the manufacturer (Illumina). All bacterial samples were sequenced on the same MiSeq run.

### 2.5. Amplification and Sequencing of the Fungal ITS Region

The fungal endophyte community was assessed by a nested PCR approach targeting the ITS region, as described previously [24,29]. In the first PCR, the primers ITS1-F_KYO2 (5′-TAG AGG AAG TAA AAG TCG TAA-3′) [30] and ITS4 (5′- TCC TCC GCT TAT TGA TAT GC-3′) [31] were used to suppress the co-amplification of plant-derived ITS regions. Obtained PCR products were subjected to nested PCR with the primer pair ITS3_KYO2 [30] and ITS4 [31] containing the MiSeq adaptors (underlined): MiSeq-ITS3_KYO2 (5′-TCG TCG GCA GCG TCA GAT GTG TAT AAG AGA CAG GAT GAA GAA CGY AGY RAA-3′) and MiSeq-ITS4 (5′-GTC TCG TGG GCT CGG AGA TGT GTA TAA GAG ACA GTC CTC CGC TTA TTG ATA TGC -3′). The PCR mixture (25 μL) contained: 5 μL of 5-fold Phusion GC buffer, 200 μM of each of the four deoxynucleoside triphosphates, 4 μM of each primer, 5% DMSO, 25 mM MgCl_2_, 0.5 U of Phusion High Fidelity DNA polymerase (Thermo Scientific), and approximately 10 ng DNA and PCR product from the first PCR as a template, respectively. The negative controls were performed using the reaction mixture without a template. The following thermal cycle scheme was utilized: initial denaturation at 98 °C for 30 s followed by 6 cycles of denaturation at 98 °C for 15 s, annealing at 53 °C for 30 s decreasing 0.5 °C in each cycle, followed by extension at 72 °C for 30 s and 29 cycles of denaturation at 98 °C for 15 s, annealing at 50 °C for 30 s, followed by extension at 72 °C for 30 s. The final extension was carried out at 72 °C for 2 min. Each sample was subjected to three independent amplifications. The negative controls were performed using the reaction mixture without template. Obtained PCR products were pooled in equal amounts, purified, and quantified as described for bacterial PCR products. The barcoding of purified fungal PCR products as well as sequencing were performed as described above for bacterial PCR products. All fungal samples were sequenced on the same MiSeq run.

### 2.6. Processing of Bacterial and Fungal Datasets

Obtained sequencing data were initially quality-filtered with the Trimmomatic tool version 0.36 [32]. Low-quality reads were truncated if the quality dropped below 12 in a sliding window of 4 bp. Subsequently, all reads shorter than 100 bp and orphan (unpaired) reads were removed. The remaining sequences were merged, quality-filtered, and further processed with USEARCH version 10.0.240 [33]. Merged bacterial reads shorter than 350 bp or longer than 550 bp were removed, while fungal reads shorter than 300 bp and longer than 500 bp were removed. Primer sequences were subsequently truncated using cutadapt (version 2.5). Reads without primer sequences as well as low-quality reads (expected error > 2) and reads with more than one ambiguous base were removed. Processed sequences of all samples were combined into a single file, and subsequently de-replicated into unique sequences. These sequences were denoised and clustered in zero-radius operational taxonomic units (zOTUs; i.e., sequences with 100% similarity) with the unoise3 algorithm [34] implemented in USEARCH [33]. Chimeric sequences were removed by the UCHIME denovo algorithm during clustering [35]. Subsequently, the remaining chimeric sequences were removed using UCHIME [35] in reference mode with the SILVA SSU Ref NR 99 132 database [36] as a reference data set for bacteria and the general release of the UNITE database version (Feb 2019) [37] for fungi. 

To assign the taxonomy of bacteria and fungi, unique and chimera-free sequences were classified using the sintax algorithm against the SILVA SSU Ref NR 99 132 database [36] and the UNITE utax reference database (Feb 2019) [37], respectively. Combined sequences of all samples were mapped on the final set of unique sequences to calculate the occurrence and abundance of each zOTU in all samples. All non-bacterial and non-fungal zOTUs were removed based on their taxonomic classification. Final zOTUs tables for bacteria and fungi are provided as Supplementary Tables S2 and S3, respectively. Sequence characteristics for prokaryotic and fungal datasets are provided as Supplementary Material Tables S4 and S5, respectively. 

### 2.7. Data Analysis

All data analyses were conducted in R version 3.6.0 [38]. Prokaryotic and fungal communities were analyzed separately. Differences were considered as statistically marginally significant with *p* ≤ 0.05 and *p* ≤ 0.1, respectively. Environmental properties were correlated by Spearman rank correlation using the cor.test function and grouped by hierarchical clustering using the hclust function. Each cluster contained those properties with a Spearman’s rho ≥ 0.9. We selected the property with the highest correlation to all other properties from the same cluster to represent the cluster. All data were normalized prior to statistical analyses. Principal Component Analysis (PCA) was performed on resemblance matrices constructed using Euclidean distance. Differences in environmental properties among the seven sites were also evaluated by Kruskal-Wallis test, followed by Dunn’s test for multiple comparisons with Benjamini–Hochberg correction using the R package FSA 0.8.25 [39]. The results of the statistical analyses are provided in Supplementary Table S4.

All alpha diversity indices were calculated 10 times and the average of all iterations was used for further statistical analyses. The zOTU tables were rarefied to 3105 (bacteria) or 2025 (fungi) sequences per sample in each iteration using the rrarefy function in vegan version 2.5–5 [40]. The diversity was calculated using the diversity function in vegan. Sample coverage was estimated using the Michaelis–Menten Fit calculated in R. For this purpose, richness and rarefaction curves were calculated using the specnumber and the rarecurve function, respectively, in picante version 1.8 [41]. The Michaelis–Menten Fit was subsequently calculated from generated rarefaction curves using the MM2 model within the drc package [42]. Good’s Coverage was calculated using the R package entropart version 1.6–1 [43] using the coverage function. Final tables containing bacterial and fungal alpha diversity, richness and sample-wise coverage are provided in Supplementary Tables S5 and S6, respectively.

The alpha diversity data were tested for normal distribution with shapiro function and for homogeneity of variance with leveneTest function using the R package car version 3.0–3 [44]. As the distribution of microbial diversity and richness significantly differed from a normal distribution, differences in alpha diversity measures among the seven sites were evaluated by Kruskal-Wallis test. Statistically significant results were followed up with Dunn’s test for multiple comparisons with Benjamini–Hochberg correction using the R-package FSA. We further tested for significant correlations between environmental properties/AMSs and alpha diversity measures by Spearman’s rank correlation using the function cor.test.

Differences in the relative abundance of the predominant fungal (≤0.5% abundance in the entire dataset) and bacterial orders (≤0.5% abundance in the entire dataset) were tested by pairwise *t*-test with Benjamini–Hochberg correction for multiple testing. The results of the statistical analysis can be found in Supplementary Table S7. Potential differences in community composition among sites and regions were investigated by permutational multivariate analysis of variance (PERMANOVA) with 1000 random permutations using the vegdist and adonis function within the vegan package [40]. Differences in community composition between the sites were tested using pairwise PERMANOVA (https://github.com/bwemheu/pairwise.adonis; version 0.1.0). Distance-based redundancy analysis (db-RDA) with forward selection of the explanatory variables using the R package vegan was performed to analyze influences of environmental properties and agroforestry management on microbial community composition. Explanatory variables were included into the model if *p* was ≤0.05. Four different dissimilarity measures were calculated in R using the vegdist function [40] and tested for the bacterial and fungal datasets: unweighted as well as weighted Bray-Curtis and unweighted as well as weighted Jaccard (binary option in the vegdist function false and true, respectively). Pre-analyses revealed that weighted Bray-Curtis dissimilarities displayed a higher environmental sensitivity based on the higher coefficients of determination. Thus, only results for this distance measure are shown here.

### 2.8. Core Community and Correlation-Based Indicator Species Analysis

To enhance the reliability of the indicator analysis, only fungal and bacterial zOTUs detected in ≥75% of the trees growing in one site and with an average relative abundance of ≥0.01% in the entire dataset were considered, hereinafter referred to as the “core” microbiome. In addition, we applied multipattern analyses using the multipatt function from the indicspecies package [45] to identify zOTUs that are highly associated with each site. The biserial coefficients (R) with a particular site were corrected for an unequal sample size using the function r.g [46]. Associated fungal and bacterial zOTUS of each site were visualized using Cytoscape version 3.6.1 [47]. The core endophyte community and the uniquely associated fungal and bacterial zOTUs are depicted in Supplementary Table S8.

### 2.9. Functional Predictions

Functional information was assigned to fungal zOTUs using FUNGuild [48]. We kept guild assignments only to those zOTUs that could be assigned with the confidence ranking of “probable” and “highly probable”, as recommended [48]. The sequence numbers of zOTUs assigned into the guilds were plotted as relative abundance (the number of sequences assigned to a specific guild divided by the number of all assigned sequences; called sequence richness). In addition, the zOTU richness was determined (the number of zOTUs assigned to a specific guild per sample divided by the number of assigned zOTUs per sample). Differences in the sequence and zOTU richness were tested by pairwise *t*-test with Benjamini–Hochberg correction for multiple testing. Final tables containing functional information for fungal endophytes and results of the statistical analyses are provided as Supplementary Tables S2 and S9, respectively. Moreover, functional profiles for bacterial communities were predicted from obtained 16S rRNA data using Tax4Fun2 [49] in reference mode (Ref100NR) with copy number correction enabled. Differences in the relative abundances of putative pathways of bacterial endophytes between sites were tested by a pairwise *t*-test with Benjamini–Hochberg correction for multiple testing.

### 2.10. Nucleotide Sequence Accession Numbers

Sequence data were deposited in the sequence read archive (SRA) of the National Center for Biotechnology Information (NCBI) under BioProject number PRJNA594470. 

## 3. Results

### 3.1. General Characteristics of the Investigated Sites

In this study, we investigated *T. cacao* leaves from different AMSs derived from five major cacao-growing regions (Obala, Boumnyébel, Bakao, Talba, Kédia) in the Central Region of Cameroon. Environmental properties measured at the different sites were correlated with each other (Supplementary Figure S1). Although some properties were significantly correlated with each other (Spearman’s rho ≤ 0.9), we included all of them in the following statistical tests. Environmental and agroforestry management predictors explained more than 80% of the variation among sites (F_6,57_ = 39.8, *p* = 0.001 ***, R^2^ = 80.7%, 999 permutations) and more than 74% of the variation among the five growing regions representing the five different AMSs (F_4,59_ = 42.9, *p* = 0.001 ***, R^2^ = 74.3%, 999 permutations).

**Figure 1:**
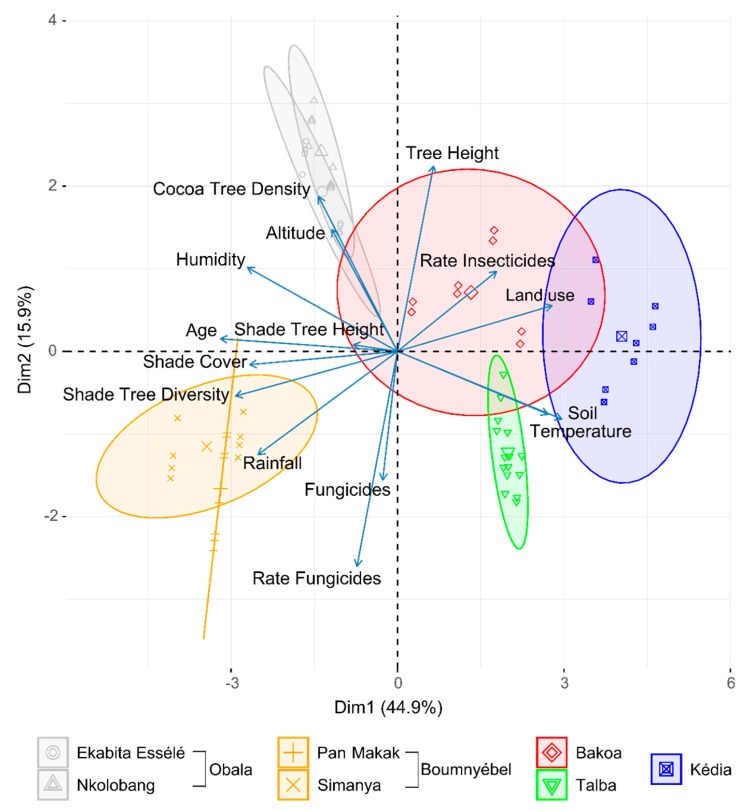
Biplot of the Principal Component Analysis (PCA) based on Euclidean distances. Comparison of the seven sampling sites in Cameroon by environmental characteristics and agroforestry management. The first two principal components (PC) explained 60.8% of total variation in the data. For further explanation on environmental data, see Supplementary Table S1.

PCA ordination (Figure 1) was used to visualize the relationship of environmental data and AMSs with sampling region. The first two principal components (PC) explained 60.8% of total variation in environmental data and agroforestry practices. The PCA showed a clear clustering of Boumnyébel and Obala samples along PC1, and portioning of Boumnyébel and Talba samples from Bakoa and Obala samples along PC2. Statistical analysis further revealed that environmental properties such as annual mean temperature or humidity as well as shade tree diversity differed significantly among the seven sites (Supplementary Tables S1 and S4). For instance, rainfall was significantly higher in Simanya compared to all other sites except Pan Makak. In addition, a significantly higher temperature in Kédia and Talba than in Ekabita Essélé, Pan Makak, Simanya and Nkolobang was recorded.

### 3.2. Sequence Characteristics

After the removal of low-quality reads, PCR artefacts (chimeras), and plant-derived contaminations, a total of 2468,643 and 2388,301 high-quality reads were obtained for fungi and bacteria, respectively (Supplementary Tables S2 and S3). Obtained sequences were assigned to 5606 fungal and 21,902 bacterial zOTUs. Sequence numbers per sample varied between 2025 and 144,753 (average 38,573) for fungi and between 3105 and 84,048 (average 37,317) for bacteria, respectively (Supplementary Tables S5 and S6). Calculated Good’s Coverage confirmed that the sampling efforts of all samples were sufficient to represent the majority of the bacterial (91.1%) and the fungal diversity (99.5%). Species accumulation curves further indicated that 82.5% of all fungal zOTUs (maximal number of zOTUs calculated = 6795) and 95.1% of all bacterial zOTUs (maximal number of zOTUs calculated = 23,036) were recovered by the surveying effort (Supplementary Figure S2). This suggests that the surveying effort was large enough to reflect the endophytic fungal and bacterial diversity in the leaves of *T. cacao.*

### 3.3. Foliar Endophyte Communities

**Figure 2:**
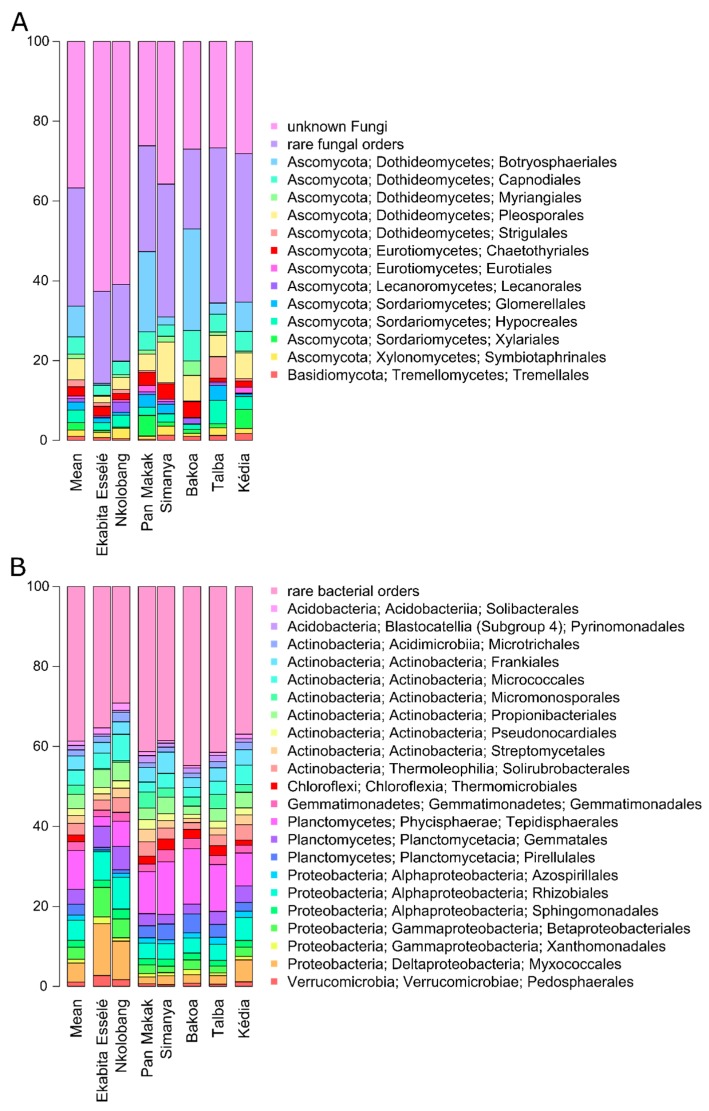
Abundant fungal (**A**) and bacterial (**B**) orders in *T. cacao* leaves collected in seven different agroforestry management systems in Cameroon. Only orders with an average abundance ≥1% (bacteria) or ≥0.5% (fungi) in the entire data set are shown.

**Figure 3:**
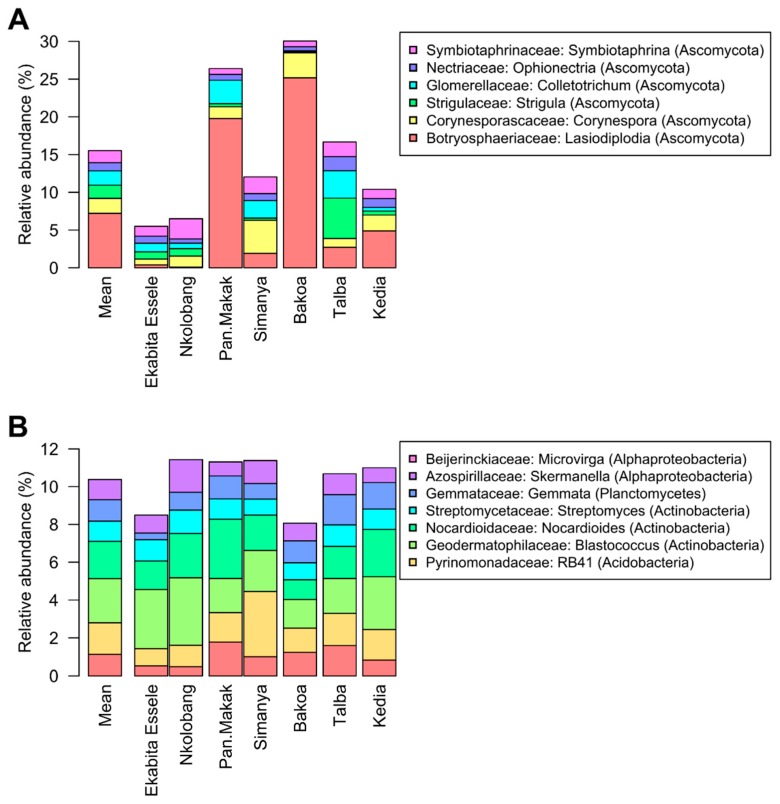
Abundant fungal (**A**) and bacterial (**B**) genera in *T. cacao* leaves collected in seven different agroforestry management systems in Cameroon. Only genera with an average abundance ≥1% in the entire data set are shown.

Fungi were represented by three abundant phyla (≥0.5% of all sequences across all samples): Ascomycota (mean abundance across all samples: 59.5%), Basidiomycota (2.8%), and Chytridiomycota (0.7%) (Figure 2A,B, Supplementary Table S2). More than one third of the fungi (mean abundance across all samples: 36.7%) were classified as unknown fungi. The predominant fungal orders in our study were Botryosphaeriales (7.7%), Pleosporales (5.3%), Capnodiales (4.3%), Hypocreales (3.1%), Chaetothyriales (2.3%), and Glomerellales (2.0%). Lasiodiplodia (7.2%) was identified as the most abundant fungal genus (Figure 3A). The three most abundant fungal zOTUs were two zOTUs of the genus Lasiodiplodia (Zotu1: L. brasiliensis; 5.9% and Zotu2: L. jatrophicola; 4.2%) as well as one unknown fungus (Zotu6: 5.0%).

Bacterial communities were dominated by Actinobacteria (mean abundance across all samples: 30.3%), Proteobacteria (22.8%), and Planctomycetes (19.7%) (Figure 3B, Supplementary Table S3). Other abundant (≥1% of all sequences across all samples) bacterial phyla were Acidobacteria (9.5%), Chloroflexi (6.8%), Gemmatimonadetes (2.7%), Verrucomicrobia (2.6%), and Bacteroidetes (1.6%). Within the Proteobacteria, Alphaproteobacteria were predominant (12.3%), followed by Gammaproteobacteria (6.0%) and Deltaproteobacteria (5.3%). The dominant bacterial orders across all samples were Tepidisphaerales (9.7%), Rhizobiales (5.1%), Myxococcales (4.8%), Micrococcales (3.8%), Gemmatales (3.7%), Propionibacteriales (3.5%), and Frankiales (3.5%). Nocardioides (2.3%) Streptomyces (2.0%) were detected as the most abundant bacterial genera (Figure 3B). At zOTU level, two zOTUs of the genera *Pseudarthrobacter* (Zotu1; 0.5%) and *Streptomyces* (Zotu2; 0.4%) were predominant.

### 3.4. Endophyte Diversity and Community Composition Per Site

Three of the dominant fungal orders (Eurotiales, Hypocreales and Botryosphaeriales) and several predominant bacterial orders differed among the seven sites (Figure 2A, Supplementary Table S7). We detected significantly higher abundances of the Botryosphaeriales in Bakoa (25.5%) leaves compared to those from Ekabita Essélé (0.4%), Nkolobang (0.2%), and Talba (2.8%). To impact of these factors on endophyte community composition, a distance-based redundancy analysis (db-RDA) based on Bray-Curtis dissimilarities was performed. In addition, we analyzed the effect of site and type of agroforestry system on the composition of bacterial and fungal endophyte communities by PERMANOVA.

**Figure 4:**
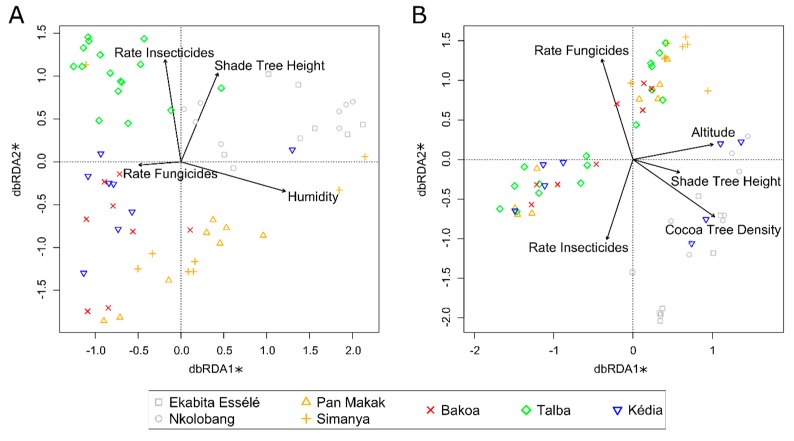
Distance-based redundancy analysis (db-RDA) plot showing the relationship of environmental properties and agroforestry management system to foliar fungal (**A**) and bacterial (**B**) endophyte communities of *T. cacao*. Ordination is based on weighted Bray-Curtis distances between samples and is color-coded by sampling region. Factors were chosen that significantly (*p* ≤0.05) contributed to the model. Axes labelled with an asterisk are significant. The first axes explained 42.5% (bacteria) or 42.2% (fungi), whereas the second axes explained 28.1% (bacteria) or 22.6% (fungi).

We observed a clear clustering of fungal (Figure 4A) and, to a lesser extent, of bacterial endophyte communities (Figure 4B), by AMS and sampling site. This is supported by the results of the PERMANOVA. Sampling site (F_(6)_ = 1.98, *p* = 0.001; R^2^ = 17.2%) and AMS (F_(4)_ = 1.95, *p* = 0.001; R^2^ = 11.7%) significantly affected the composition of bacterial communities. Similarly, the composition of fungal endophytes differed significantly between sites (F_(6)_ = 2.26, *p* = 0.001; R^2^ = 19.2%) and agroforestry systems (F_(4)_ = 2.83, *p* = 0.001; R^2^ = 16.1%). The best-fit explanatory variables for fungal community composition were insecticide rates, fungicide rates, humidity, and shade tree height (F_5,58_ = 2.31, *p* = 0.001; Figure 4A, Table 2). The community composition of bacterial endophytes was significantly affected by insecticide rate, fungicide rate, shade tree height, altitude and cacao tree density (F_6,57_ = 2.30, *p* = 0.001; Figure 4B, Table 2). Multiple comparisons revealed that the composition of fungal communities differed significantly among all AMSs, while bacterial community composition in leaves from Obala differed from those collected in Talba, Kédia, and Bakoa (*p* ≤ 0.05).

In addition to changes in community composition, we analyzed differences in alpha diversity measures among sites and AMSs. Richness (number of observed unique sequences) and diversity (Shannon diversity index H’) for fungal communities varied between 71 and 443 and between 1.32 and 5.34, respectively (Table 3 and Supplementary Table S5). Bacterial richness and diversity ranged from to 1206 to 2096 and from 6.13 to 7.44, respectively (Supplementary Table S6). Bacterial alpha diversity measures did not differ among the sites (richness: *p* = 0.79; diversity: *p* = 0.43) and the agroforestry system types (richness: *p* = 0.75; diversity: *p* = 0.32) (Table 3). In contrast, fungal richness (*p* < 0.001) and diversity (*p* = 0.001) differed among the five agroforestry systems, with, site significantly affecting fungal richness (*p* = 0.003) and diversity (*p* = 0.006).

Multiple comparisons revealed a significantly higher fungal diversity and richness in Boumnyébel, Talba, and Kédia compared to Obala. Temperature and fungicide rate were positively correlated with fungal richness and diversity, while humidity, cacao tree density, and tree height were negatively correlated with fungal richness and/or diversity (Table 4).

### 3.5. Bacterial and Fungal Taxa Associated with Agroforestry System Type

To identify bacterial and fungal core taxa associated with sites/agroforestry management systems, we performed an indicator species analysis. The core zOTUs were selected based on their relative frequency (≥75% occurrence in each of the seven sites) (Supplementary Table S8). The core endophyte community was represented by ten fungal zOTUs (0.2% of all fungal zOTUs), such as *L. brasiliensis* (Zotu1; mean abundance across all samples: 5.9%), *Colletotrichum hymenocallidis* (Zotu22; 0.8%), and *Ophionectria trichospora* (Zotu29; 0.9%). Moreover, we detected 199 bacterial zOTUs (0.9% of all bacterial zOTUs), including eleven zOTUs belonging to the genera *Streptomyces* and *Blastococcus* and nine zOTUS of the genus *Nocardioides.* A total of 20.1% of all fungal and 12.6% of all bacterial sequences were assigned to these core zOTUs.

**Figure 5:**
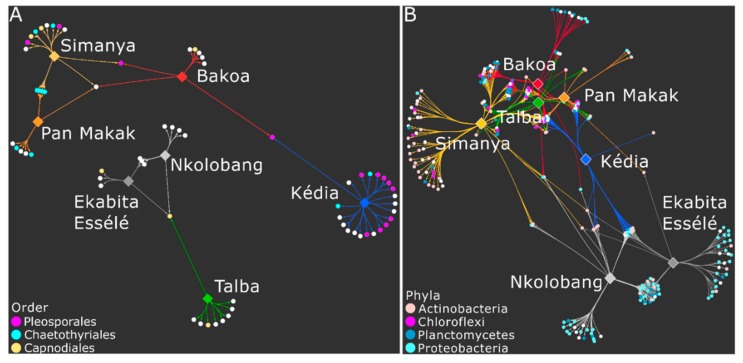
Bipartite association network of fungal (**A**) and bacterial (**B**) zOTUs in *T. cacao* leaves significantly associated with site. The sites are color-coded as in Figure 1. Bacterial phyla and fungal orders, which were predominant in the dataset and/or in one site, are highlighted.

The indicator species analysis further identified those significantly associated fungal and bacterial zOTUs using the following threshold levels: sample prevalence (≥75% in one site) and relative abundance (≥0.01% in the entire dataset). We detected a higher number of significantly associated bacterial (*n* = 523) than fungal (*n* =79) zOTUs (Figure 5, Supplementary Table S8). The lowest and highest number of significantly associated bacterial zOTUs were found in Pan Makak (*n* = 51) and Simanya (*n* = 118), respectively. Most bacterial zOTUs belonged to the genera Pir4 lineage (*n* = 19), *Nocardioides* (*n* = 12), *Actinoplanes* (*n* = 12), Candidatus *Alysiosphaera* (*n* = 11), *Marmoricola* (10), *Micromonospora* (*n* = 9), *Blastococcus* (*n* = 8), and *Krasilnikovia* (*n* = 8). For fungi, the lowest and highest number of significantly associated zOTUs were detected for Ekabita Essélé (*n* = 7) and Kédia (*n* = 22), respectively.

### 3.6. Fungal Functionality Differs between the Agroforest Management Systems

**Figure 6:**
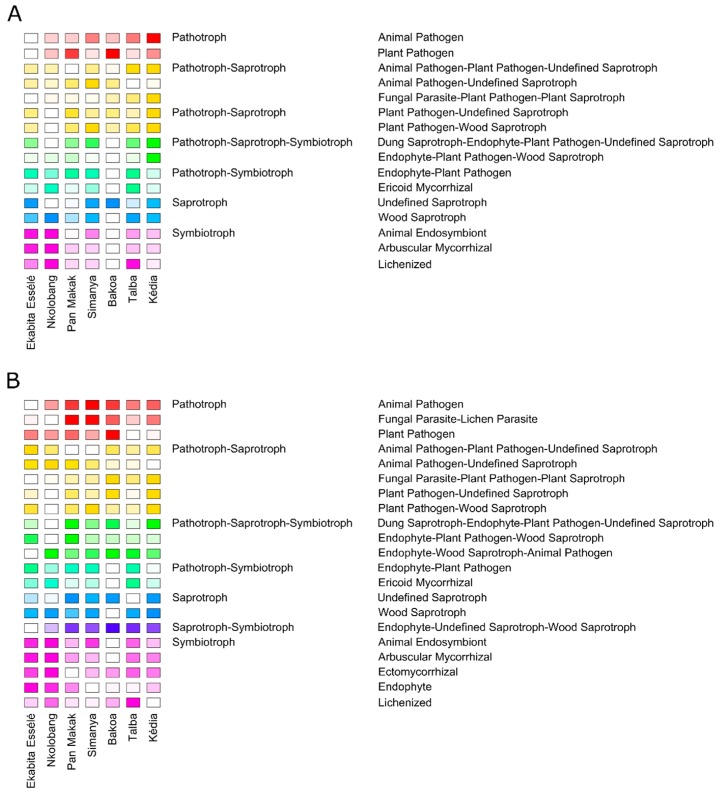
Functional guilds of fungal endophytes in *T. cacao* leaves. The relative abundance of fungal sequences (sequence richness) per guild (**A**) and the proportion of zOTUs (zOTU richness) per guild (**B**) is shown for the seven sites. The analyses are based on 31.5% of the zOTUs (*n* = 1766 zOTUs). The sequence richness and zOTU richness were calculated by the number of sequences assigned to a specific guild divided by the number of all assigned sequences and by the number of zOTUs assigned to a specific guild per sample divided by the number of zOTUs per sample, respectively. Following the suggestion of Nguyen et al. [48], we combined both dimensions (sequence and zOTU richness) to better reflect the relative importance of fungal life strategies in an environment. Only guilds with an average abundance ≥0.5% in the entire data set are shown.

We also analyzed functional changes in fungal endophytes with respect to AMS. For this purpose, functional guilds of endophytic fungi were determined using FUNGuild [48]. In total, highly probable and probable life strategies for 1766 of the 5606 zOTUs (= 31.5%) were predicted (Supplementary Table S9). We identified more abundant (≥0.5% across all samples) functional guilds by investigating the zOTU richness (*n* = 21) compared to sequence richness (*n* = 16) (Figure 6; Supplementary Table S2). Most of the fungal sequences were classified as undefined saprotrophs (sequence richness: 24.2% mean abundance across all samples; zOTU richness: 20.2.%). Other abundant functional guilds were plant pathogens (sequence richness: 23.8%; zOTU richness: 13.5%) and animal endosymbionts (sequence richness: 7.4%; zOTU richness: 14.8%). However, we observed differences in the sequence richness among sites. Undefined saprotrophs were predominant in Simanya (28.3%), Talba (20.7%), and Ekabita Essélé (29.5%), whereas lichenized fungi dominated in Nkolobang (21.7%) (Figure 6A). Plant pathogens were the dominant functional guild in Pan Makak (35.8%), Kédia (26.4%), and Bakoa (43.3%). Analysis of the zOTU richness revealed that undefined saprotrophs were the most abundant functional guild in Kédia (24.1%), Pan Makak (25.1%), Simanya (22.1%), Bakoa (24.1%), and Ekabita Essélé (18.0%) (Figure 6B). Animal endosymbionts were predominant in Nkolobang (19.3%) and lichenized fungi in Talba (17.1%). 

Multiple comparisons revealed that two functional guilds (animal pathogen-plant pathogen-undefined saprotrophs and wood saprotrophs) did not differ in both sequence and zOTU richness among sites (Figure 6). Moreover, the sequence richness of most functional guilds, including animal pathogens, ericoid mycorrhizal, plant pathogens, or endophyte-plant pathogens did not differ between the sites (Figure 6A). In contrast, we observed a significantly higher sequence richness of the functional guild of arbuscular mycorrhiza in Ekabita Essélé (2.6%) and Nkolobang (3.0%) compared to the other sites (≤0.8%). The zOTU richness showed that arbuscular mycorrhiza were significantly more abundant in Ekabita Essélé (3.8%) and Nkolobang (4.2%) than in Bakoa (0.9%) (Figure 6B). We also observed a significantly lower zOTU richness for animal pathogens in Ekabita Essélé (0.9%) compared to all other sites (≥2.5%) except Nkolobang (2.1%). A significantly higher zOTU richness of plant pathogens was observed in Bakoa (17.6%) compared to Kédia (11.5%) and Talba (11.3%). Lichenized fungi were more abundant in Talba (17.1%) compared to all other sites (≤12.9%).

### 3.7. Predicted Functional Profiles of Bacterial Endophytes

**Figure 7:**
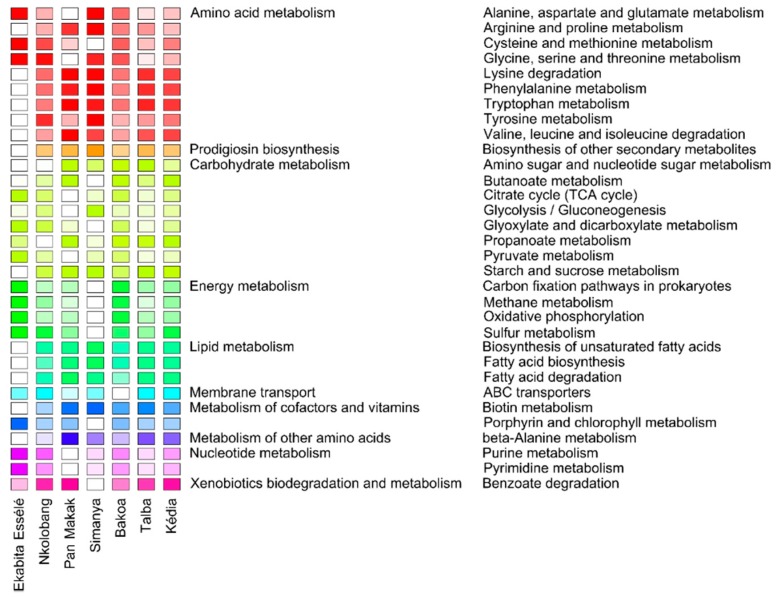
Predicted functional profiles of bacterial endophytes in *T. cacao* leaves. The color code refers to the relative abundance of each pathway, with high predicted abundances (dark-colored) and low predicted abundances (light-colored). The relative abundances of pathways were calculated for each site. Only pathways with an average abundance ≥1% in the entire data set are shown.

To investigate potential changes in bacterial community function between the AMSs, functional profiles for bacterial endophytes were predicted from 16S rRNA gene data using Taxa4Fun2 [49]. Approximately 82% of all zOTUs, representing 76.7% of all sequences obtained, were used in the prediction (Supplementary Table S9). We focused on important pathways involved in metabolism, environmental information processing and organismal systems, resulting in 32 abundant pathways (≥1% of all sequences across all samples) (Figure 7). The highest abundances were observed for ABC transporters (mean abundance across all samples: 11.1%), Valine, leucine, and isoleucine degradation (2.8%), propanoate metabolism, fatty acid degradation, pyruvate metabolism, and butanoate metabolism (all: 2.5%). The three pathways with the lowest mean abundance of the 32 selected pathways across all samples (1.1%) were tyrosine metabolism, porphyrin and chlorophyll metabolism, and the citrate cycle (TCA cycle).

The majority (*n* = 22) of the 32 abundant pathways, such as pathways involved in the metabolism of cofactors and vitamins, pathways involved in both lipid and energy metabolism (excluding sulfur metabolism), as well as ABC transporters, did not differ in their relative abundance among the sites (Figure 7). In contrast, we observed significantly higher abundances of pyrimidine metabolism, purine metabolism, glyoxylate and dicarboxylate metabolism, as well as sulfur metabolism in Ekabita Essélé compared to Pan Makak, Simanya, and Talba. We also found significantly higher abundances of starch and sucrose metabolism in Pan Makak, Simanya, Talba, and Kédia than in Ekabita Essélé and higher abundances of the tyrosine metabolism in Simanya compared to all other sites. Significantly lower abundances of arginine and proline metabolism as well as phenylalanine metabolism were observed in Ekabita Essélé than in Simanya, whereas the opposite was detected for cysteine and methionine metabolism.

## 4. Discussion

To our knowledge, this is the first metabarcoding study simultaneously investigating the bacterial and fungal endophytes of *T. cacao* trees and their response towards the agroforestry system type. The leaves were colonized by a high diversity of fungal species; however, more than one third of these fungal zOTUs could not be further classified. These results indicate that the identity and ecology of many fungal endophytes in these regions remain largely uncharacterized. The large proportion of Ascomycota and comparatively few Basidiomycota generally agrees with previous studies on foliar fungal communities from tropical and temperate trees [50,51,52,53]. Two of the dominant fungal zOTUs belonged to *Lasiodiplodia*, a genus of the Botryosphaeriaceae. This family contains numerous fungal species, which are able to infect a diverse range of host plant species or known to live as saprophytes or endophytes within seeds and other living plant tissues [54,55].

The taxonomic composition of bacterial endophytes in *T. cacao* leaves differed from previous studies on bacterial endophytes of temperate tree species [51,56]. For instance, higher abundances of Proteobacteria were recorded in a recent study on Maple trees in Germany. Nonetheless, our results are in line with a previous study on bacterial communities in the roots of the tropical tree species *Eucalyptus urograndis* [57] and the leaves of several woody species [58]. Two zOTUs of the bacterial genera *Pseudarthrobacter* and *Streptomyces* (both Actinobacteria) were predominant in our samples. This is noteworthy as members of the Actinobacteria, especially the genus *Streptomyces*, are known to produce a wide range of antimicrobial compounds [59,60].

The composition of fungal and bacterial endophyte communities in *T. cacao* leaves differed among the sampling sites and AMSs. The observed differences in the relative abundances of the predominant fungal and bacterial orders most likely arose from differences in management practices and environmental properties in the studied cacao agroforestry systems. This is in line with previous studies investigating endophyte communities from tree species, analyzed from different locations [13,51,61]. We further recorded that fungal but not bacterial endophyte alpha diversity measures differed among sites. Contrarily, site significantly affected bacterial but not fungal alpha diversity values in leaves of two *Acer* species [51]. In addition, AMS affected fungal richness and diversity but not bacterial alpha diversity. Köberl et al. [62] reported neither a significant impact of agroforestry type nor biogeography on the gammaproteobacterial diversity in leaves of *Musa* spp. [62]. In another study on fungal endophytes in the leaves of *B. pendula* grown in natural and managed boreal forests, silvicultural practices affected the species composition and endophyte frequencies of these fungi [63]. The authors proposed that the observed differences reflect the mode of fungal spreading and largely depend on biotic and abiotic environmental conditions determining the abundance of infection sources and the success of transmission and germination of specific fungal endophyte species. This hypothesis is supported by our study as environmental properties, such as temperature and/or humidity as well as agroforestry practices influenced alpha diversity measures and the community composition of fungal endophytes. Previous studies on the fungal endophyte communities of trees in Hawaiian landscapes [64], the leaves of Olea europaea in the Mediterranean area [65], and the leaves of Ficus tree species in the Philippines [52] also found that patterns of community similarity are strongly associated with rainfall and elevation: 

Consistent with previous studies on fungicide effects on leaf-associated microbial communities [26,66,67], insecticide and fungicide application rate affected the composition of both fungal and bacterial endophytes in leaves of *T. cacao*. Interestingly, fungicide rate was positively correlated with fungal richness and diversity. In contrast, fungicide treatment had no effect on fungal endophyte diversity in *A. altissima* [26]. We speculate that the fungicide metalaxyl, used in the site sampled is highly selective against some fungi [68]. This might have created free niches in the leaf tissues subsequently colonized by other fungi.

Fungal and bacterial endophyte community composition as well as fungal richness and diversity in leaves collected in Obala significantly differed from those collected in Talba, Kédia, and Bakoa, respectively. As favorable growing conditions, such as higher humidity and temperature or lower UV radiation, can influence the rate and extent of fungal colonization and survival on leaf surfaces [65,69], we hypothesize that environmental conditions (e.g., microclimate and/or light intensity) in Obala have prevented fungal endophyte colonization. Scholtysik et al. [70] found that composition of endophytes in sun-exposed leaves from the top of full-grown *Fraxinus excelsior* trees differed considerably from leaves in the shade crowns and in the understorey. Lastly, we hypothesize that our results are related to the significantly higher cacao tree density observed in Obala or differences in shade tree height among the sites, as shade tree height and cacao tree density were main drivers of bacterial and/or fungal endophyte community composition. 

Fungal and bacterial endophytes responded differently towards the factors investigated. These results are consistent with previous studies on plant-associated microbial communities [14,51] and are attributed to differences in lifestyle strategies (i.e., colonization behaviour) of fungal and bacterial endophytes [10,71]. Another possible explanation is that fungal endophytes might be more sensitive to agroforestry system type and/or environmental properties than bacteria. Due to the limitations in the study design, future studies investigating more agroforests at different cacao growing regions are needed to better understand causal factors influencing bacterial and fungal endophyte communities.

Our analysis revealed that leaves of *T. cacao* formed a core endophyte microbiota of a few fungal and 199 bacterial zOTUs, although study sites were hundreds of kilometers apart. There are several potential explanations for this results. Firstly, it is possible that the core community bacterial and fungal taxa colonized tree leaves across cacao plantations by dispersal through numerous vectors (air, rain, insect vectors), homogenizing the endophyte community as suggested for the phyllosphere communities of several tree species [72]. Another possible explanation is that some bacteria and fungi are obligate endophytes and are thus restricted to a life inside of plant tissues [73,74]. These obligate endophytes might constitute larger parts of the core community of *T. cacao* leaves. Several fungal zOTUs identified here, such as *L. brasiliensis* [75] or *C. hymenocallidis* [76], have been observed as plant pathogens, while *O. trichospora* is a relatively common species occurring on rotting wood in tropical regions [77]. It is likely that the above-mentioned fungi are latent pathogens and latent saprotrophs [78], as *T. cacao* leaves did not show any symptoms of diseases. Vega et al. [79] suggested that some endophytic fungi, such as *Colletotrichum* are either ubiquitous in coffee-growing regions because of the exchange of *Coffea* plants and seeds or because of intrinsic factors (i.e., the global distribution of the fungi themselves). These reasons might also have played a role in the current study. Lastly, it could be that the observed fungal and bacterial endophytes in the core community are seed-borne, as described for fungi such as *L. theobromae* [80] or bacteria [81]. As we did not investigate the seedling or seed endophytes of *T. cacao*, the transmission modes and colonization routes of fungal and bacterial endophytes await further research. 

We further recorded that the cacao agroforests showed uniquely associated fungal and bacterial zOTUs, suggesting specific adaptions to environmental properties and agroforestry practices in the respective sites. Another explanation is that the *T. cacao* trees select for beneficial microorganisms as they provide an advantage for their host plant [73,82]. Some of the uniquely associated bacterial zOTUs, including *Streptomyces*, *Actinoplanes*, or *Micromonospora*, are known to have plant growth-promoting abilities. *Actinoplanes* spp. are a group of filamentous bacteria that can parasitize *Pythium* spp. or related fungi [83]. As mentioned above, *Streptomyces* are best-known for their wide range of produced biomolecules, which in turn might be excellent agents for controlling various fungal and bacterial phytopathogens [59]. Similarly, some species of the *Micromonospora* produce antimicrobial and antifungal compounds that act to protect plants from pathogens (reviewed in [84]). In addition, we identified animal-associated fungal species. *C. aphidis* usually grows on aphids and superficially on leaves attacked by aphids [85] and *S.* *buchneri* was isolated from the beetle *Stegobium paniceum* that fed on the pulverized fruits of Capsicum [86]. The authors hypothesized that the life cycle of this species includes a symbiotic phase in the gut of the beetle and an unknown sexual morph growing on plant substrates.

We applied FUNGuild [48] and Tax4Fun2 to assess the functional responses of fungal and bacterial endophytes towards agroforestry practices as well as environmental conditions. Consistent with two recent studies on root-associated fungal communities in the Bolivian Andes [87] and aerial fungal endophytes of three grass species in Germany [24], saprophytic fungi dominated fungal endophyte communities. We identified more abundant functional guilds when investigating the zOTU richness compared to sequence richness. This supports the suggestion of Nguyen et al. [48] that combining both dimensions reflect the relative importance of fungal life strategies in an environment. The high abundance of ABC transporters in the predicted functional profile of bacterial endophytes might be related to the plant-associated lifestyle of endophytes, which requires the efficient uptake of plant synthesized nutrients [88]. Similarly, Hardoim et al. [10] suggested that regulatory genes related to the stoichiometry of carbon and nitrogen metabolism and those involved in the metabolism of vitamins and nucleotides and in stress responses are of fundamental importance for a life inside plants. 

We detected that several fungal functional guilds and predicted pathways of bacterial endophytes differed in their relative abundances among the sites and agroforestry system types. These results might be related to differences in agroforestry practices and environmental properties, which altered community composition and conseqeuntly community functioning. The functional changes of fungal and bacterial endophytes towards agricultural practices have been observed previously [11,24,67].

It has been proposed that the core microbiome might be functionally significant for the host plant, while the accessory microbiome is expected to contain more dispensable functions or microorganisms whose presence is related to interactions with the surrounding environmental conditions [82]. We thus assume that functional guilds and predicted bacterial pathways that did not differ between the sites are related to the core microbiome, while the uniquely associated endophytes are responsible for observed functional changes. However, only a low number of fungal and bacterial zOTUs and sequences could be used in the analyses, as the ecological role of most microorganisms in the plant endosphere and their functions remain still unknown [10,71]. Consequently, further studies are needed to better understand how management regimes affect functional traits of bacterial and fungal endophyte communities and their functioning in leaves of the economically important tree species *T. cacao.*

## 5. Conclusions

To date, studies on fungal and bacterial endophytes in *T. cacao* trees growing in different agroforestry systems are lacking. In the current study, we applied large-scale metabarcoding to assess compositional and functional responses of fungal and bacterial endophytes in the leaves of *T. cacao* trees growing in five major cacao-growing regions in the Central Region of Cameroon. The diversity and richness of fungal but not bacterial endophytes differed among the five cacao regions, suggesting that fungal alpha diversity is more sensitive to agroforestry system type and/or environmental properties than bacterial alpha diversity. Our results further suggest that bacterial and fungal endophyte community composition are affected predominantly by agroforestry practices and, to a lesser extent, by environmental properties. The correlation-based indicator species indicated that the core microbial community forms stable associations with *T. cacao* across geographic scales. Functional analyses, based only a minor part of microbial zOTUs and sequences, revealed that several predicted pathways of bacterial endophytes and the functional guilds of fungal endophytes differed between the agroforests, which might be attributed to several bacteria and fungi specifically associated with a single agroforest.

## Figures and Tables

**Table 1:** Landscape characteristics of the five regions Boumnyébel (sites Pan Makak, Simanya), Talba, Bakoa, Kédia and Obala (sites Ekabita Essélé, Nkolobang). See Supplementary Table S1 for further details.

Region	Plantation	Trees	Agroforestry Management	Agricultural Land	Forest Land
Boumnyébel	Pan Makak 1	4	extensively managed old (cocoa is grown under a dense cover of shade tree species)	20% cocoa fields, 10% annual crop	70% pristine forest, with forest reserve
	Pan Makak 2	4			
	Simanya 1	4			
	Simanya 2	4			
Talba	Talba 1	4	intensively managed manure (cocoa is grown in larger farms)	70% cocoa fields, 5% annual field crops	25% pristine forest, no reserve
	Talba 2	4			
	Talba 3	4			
	Talba 4	4			
Bakoa	Bakoa 1	2	extensively managed young (cocoa is grown on modified savannah agroecosystems)	50% cocoa fields, 25% annual field crops, 5% patchy pasture fields	20% secondary forest, no reserve
	Bakoa 2	2			
	Bakoa 3	2			
	Bakoa 4	2			
Kédia	Kédia 1	2	intensively managed young (cocoa is grown under full sun)	65% cocoa fields, 25% annual field crops, 5% pasture lands	5% secondary forest
	Kédia 2	2			
	Kédia 3	2			
	Kédia 4	2			
Obala	Ekabita Essélé 1	4	home garden cacao forest (cocoa is grown nearby houses with a high variety of fruit tree species)	70% cocoa fields, 25% annual crop fields of mixed crops, agroforestry trees	5% secondary forest, no forest reserve
	Ekabita Essélé 2	4			
	Nkolobang 1	4			
	Nkolobang 2	4			

**Table 2:** Results of the db-RDA that describes the effect of environmental properties and agroforestry management systems on endophyte community composition in *T. cacao* leaves.

	DF	SumOfSqs	F	*p* Value	
Bacteria					
Entire Model	6	3.89	2.30	0.001	***
Cocoa Tree Density	1	0.49	1.72	0.024	*
Altitude	1	0.70	2.47	0.002	**
Fungicides rate/Cropping Season	1	0.51	1.82	0.022	*
Fungicides	1	0.70	2.49	0.002	**
Shade Tree Height	1	0.56	1.97	0.013	*
Insecticides rate/Cropping Season	1	0.45	1.58	0.049	*
Residual	57	16.08			
Fungi					
Entire Model	5	4.33	2.31	0.001	***
Humidity	1	1.05	2.78	0.001	***
Shade Tree Height	1	0.70	1.87	0.006	**
Fungicides rate/Cropping Season	1	0.71	1.90	0.005	**
Fungicides	1	0.58	1.54	0.019	*
Insecticides rate/Cropping Season	1	0.55	1.48	0.042	*
Residual	58	21.77			

For each model, forward selection was applied to identify which factors best described variation in community composition using an inclusion threshold of α = 0.05 and Bray-Curtis distances. Marginal effects of terms are shown (i.e., terms were not added sequentially). The number of unrestricted permutations: 999. Significance level: * *p* ≤ 0.05, ** *p* ≤ 0.01, *** *p* ≤ 0.001. SumOfSps: sum of squares, DF: degrees of freedom.

**Table 3:** Alpha diversity measures (mean ± standard deviation) for bacterial and fungal endophytes in leaves of *T. cacao.* Richness and diversity are represented by the number of observed zero-radius operational taxonomic units (zOTUs) and Shannon diversity index H’, respectively.

	Fungi		Bacteria	
	Richness	Diversity	Richness	Diversity
Obala	178 ± 76^A^	3.04 ± 0.98^A^	1729 ± 218	7.02 ± 0.31
- Ekabita Essélé	174 ± 72^a^	2.85 ± 0.98^a^	1672 ± 255	6.92 ± 0.4
- Nkolobang	182 ± 85^a^	3.24 ± 0.99	1785 ± 172	7.11 ± 0.18
Boumnyébel	279 ± 106^B^	4.09 ± 1.31^B^	1820 ± 85	7.17 ± 0.1
- Pan Makak	250 ± 110^b^	3.96 ± 1.47	1814 ± 88	7.16 ± 0.09
- Simanya	307 ± 101^b^	4.22 ± 1.23	1826 ± 87	7.19 ± 0.1
Bakoa	231 ± 82^AB^	3.48 ± 1.11^AB^	1789 ± 160	7.15 ± 0.19
Talba	292 ± 55^Bb^	4.44 ± 0.41^Bb^	1804 ± 137	7.17 ± 0.13
Kédia	330 ± 87^Bb^	4.42 ± 0.64^B^	1782 ± 101	7.12 ± 0.11

^A,B^ Different superscript letters indicate significant differences (*p* ≤  0.05) between the five regions (Obala, Boumnyébel, Bakoa, Talba and Kédia). Note that fungal diversity differed marginally (*p* ≤ 0.1) among Bakoa and Talba/Boumnyébel. In addition, fungal richness differed marginally between Bakoa and Kédia. ^a,b^ Different superscript letters indicate significant differences (*p* ≤  0.05) between the seven sites (Ekabita Essélé, Nkolobang, Pan Makak, Simanya, Bakoa, Talba and Kédia). Note that there were marginally significant differences in fungal diversity between Ekabita Essélé and Pan Makak/Simanya/Kédia, and between Nkolobang and Talba.

**Table 4:** Correlation between alpha diversity measures and environmental properties/an agroforestry management system based on Spearman’s rank correlation. Richness and diversity are represented by the number of observed zOTUs and Shannon diversity index H’, respectively.

Tested Variable		Fungal Endophytes				Bacterial Endophytes			
		Richness		Diversity		Richness		Diversity	
Environmental properties	DF	rho	p	rho	p	rho	p	rho	p
Altitude	62	−0.11	0.37	−0.10	0.43	0.14	0.27	0.08	0.56
Temperature	62	0.34	0.006	0.26	0.04	−0.06	0.65	−0.01	0.92
Humidity	62	−0.34	0.006	−0.26	0.04	0.02	0.87	−0.11	0.41
Rainfall	62	0.12	0.34	0.24	0.052	0.04	0.77	0.04	0.73
Agroforestry management									
Age	62	−0.22	0.07	−0.11	0.39	0.05	0.70	0.02	0.87
Cacao Tree Density	62	−0.26	0.04	−0.21	0.099	−0.09	0.46	−0.18	0.16
Insecticides rate *	62	−0.06	0.62	−0.05	0.70	−0.08	0.51	−0.09	0.49
Fungicides rate *	62	0.29	0.02	0.42	<0.001	0.12	0.34	0.17	0.18
Shade Tree Height	62	−0.14	0.26	0.004	0.97	−0.18	0.16	−0.17	0.18
Shade Tree Diversity	62	−0.07	0.57	0.04	0.78	0.14	0.28	0.09	0.48
Shade Cover	62	−0.18	0.16	−0.07	0.58	0.02	0.86	0.02	0.88
Tree Height	62	−0.27	0.03	−0.29	0.02	−0.03	0.83	−0.02	0.85

Statistically significant (*p* ≤ 0.05) and marginally significant (*p* ≤ 0.1) *p* values are written in bold and are underlined, respectively. DF: degrees of freedom. * Fungicides and Insecticides rate/Cropping Season.

## Supplementary Materials

The following are available online at https://www.mdpi.com/2076-2607/8/3/405/s1.
