# Supplementary material for: Agroforestry Management Systems Drive the Composition, Diversity, and Function of Fungal and Bacterial Endophyte Communities in Theobroma Cacao Leaves
Source: Microorganisms. 2020 Mar 13;8(3):405. doi: 10.3390/microorganisms8030405 (PMC7143032; doi:10.3390/microorganisms8030405)
Supplement: Supplementary file 1 [file microorganisms-08-00405-s001.zip › Figure_S1.docx]

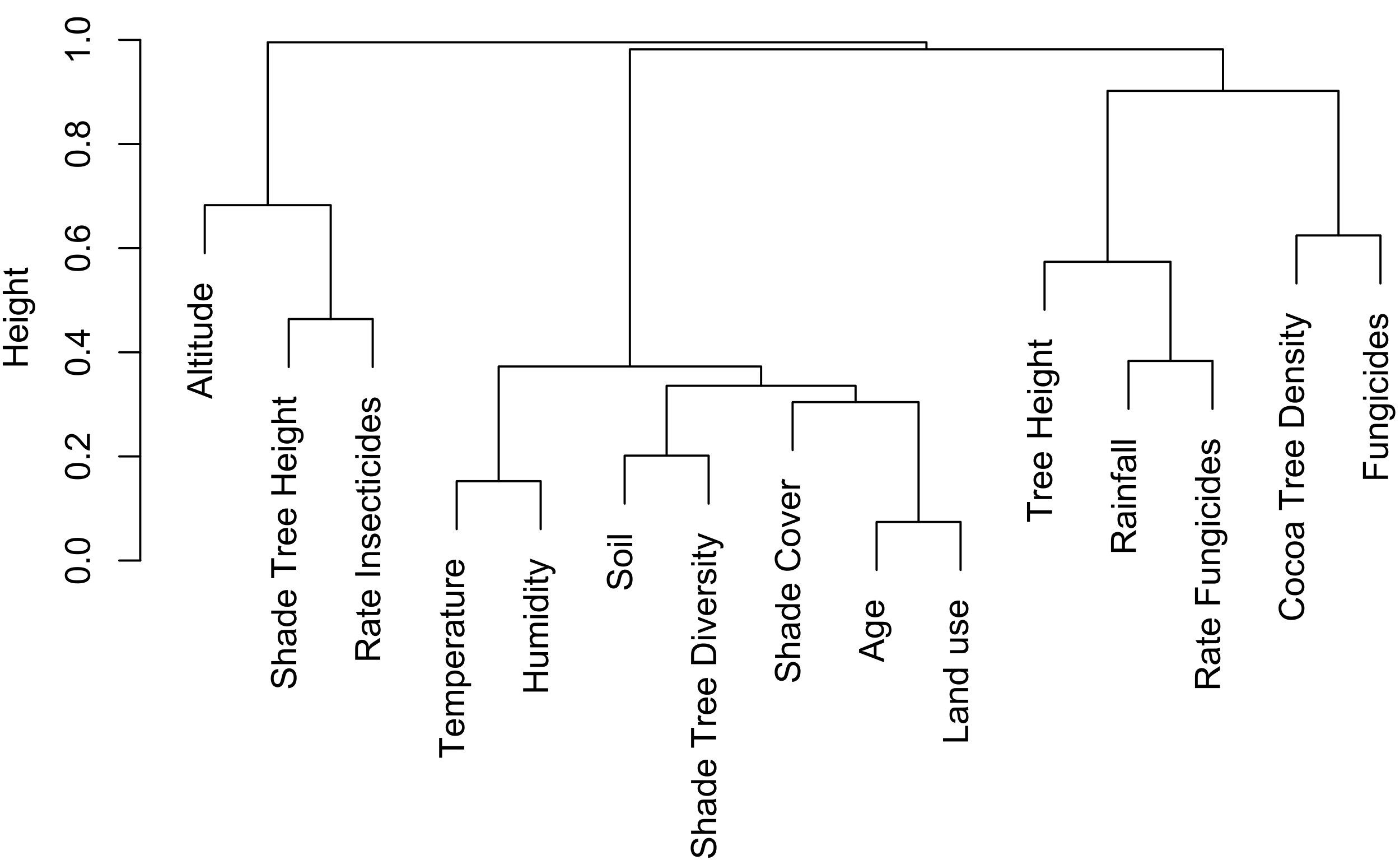


Figure S1. Cluster dendrogram for environmental characteristics and agroforestry management systems based on Spearman rank correlation. For further explanation on environmental data see Supplementary Table S1.
