# Supplementary material for: Agroforestry Management Systems Drive the Composition, Diversity, and Function of Fungal and Bacterial Endophyte Communities in Theobroma Cacao Leaves
Source: Microorganisms. 2020 Mar 13;8(3):405. doi: 10.3390/microorganisms8030405 (PMC7143032; doi:10.3390/microorganisms8030405)
Supplement: Supplementary file 1 [file microorganisms-08-00405-s001.zip › Figure_S2.docx]

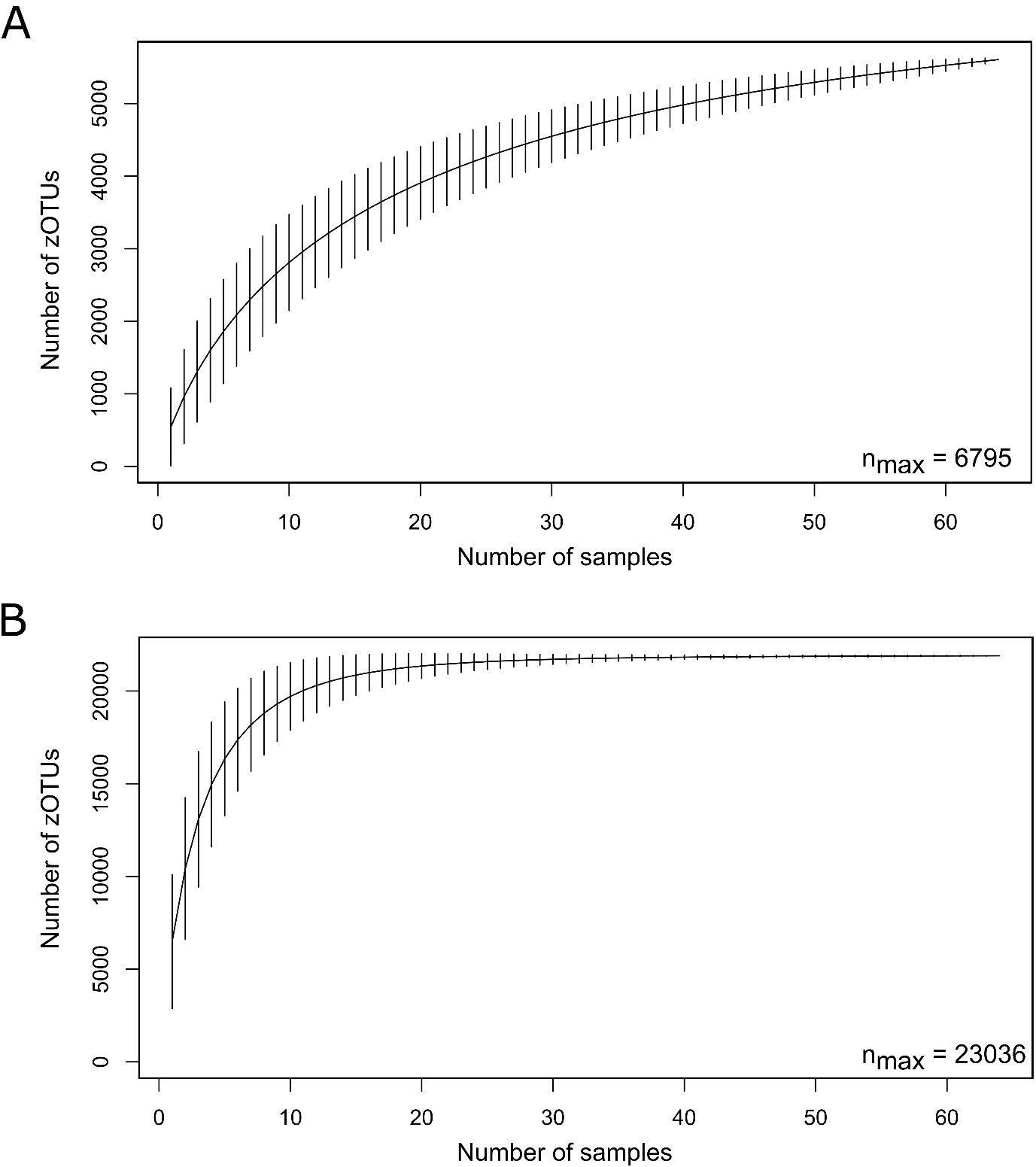


Figure S2. Species accumulation curves for fungal (A) and bacterial (B) endophytes in *T. cacao* leaves. The maximal number of zOTUs (n_max_) was calculated based on Michaelis-Menten Fit.
